# Unveiling the qualities of a ‘good doctor’: family carers’ and healthcare professionals’ perspective on dementia healthcare in India

**DOI:** 10.1186/s12939-025-02408-3

**Published:** 2025-02-17

**Authors:** Upasana Baruah, Rachita Rao, Josefine Antoniades, Santosh Loganathan, Mathew Varghese, Claudia Cooper, Mike Kent, Briony Dow, Bianca Brijnath

**Affiliations:** 1 Aged Care and Social Gerontology Division, National Ageing Research Institute, Parkville, VIC Australia; 2 Department of Psychiatry, National Institute of Mental Health and Neurosciences, Bengaluru, Karnataka India; 3 School of Humanities and Social Science, La Trobe University, Bundoora campus Plenty Road, Bundoora, VIC 3086 Australia; 4 National Ageing Research Institute, Parkville, VIC Australia; 5 Department of Psychiatry, St John’s Medical College, Bengaluru, Karnataka India; 6 Wolfson Institute of Population Health, Queen Mary University of London, London, UK; 7 School of Media, Creative Arts and Social Inquiry, Curtin University, Perth, WA Australia; 8 Faculty of Dentistry, Medicine and Health Sciences, University of Melbourne, Melbourne, VIC Australia

**Keywords:** Dementia care, Good doctor, Multidisciplinary care team, India, Healthcare professionals, Person-centered care, Doctor-patient relations, Health services accessibility

## Abstract

**Background:**

The escalating prevalence of dementia in India highlights the need for effective dementia care, particularly in a context marked by limited specialized services and resources. In response to this growing challenge, we sought to contribute to the understanding of societal expectations of multidisciplinary dementia care by exploring the qualities that family carers and healthcare professionals value in dementia care professionals within a multidisciplinary team in India.

**Methods:**

The aim of the study was to describe the perspectives of carers and healthcare professionals regarding the attributes of a ‘good doctor’ in the context of accessing care for individuals with dementia in India. The research involved qualitative face-to-face interviews with 19 family carers and 25 healthcare professionals in Bengaluru, India, with data collected between March and July 2022.

**Results:**

Using a thematic analysis framework, four main themes emerged: [1] accessibility and availability [2], empathetic engagement and effective communication [3], knowledge and competency, and [4] systemic reforms and culturally competent multilevel support. Public and professionals’ perceptions of ‘good’ care appeared to have shifted from seeking cures to prioritizing time, counselling, and information, reflecting a more holistic understanding of support needed. Interviewees valued interactions in which they perceived practitioners acting with patience, compassion, respect for dignity of the person with dementia, and professional competence. Effective communication was key. Challenges in accessing quality dementia care included inadequate infrastructure, lack of specialized services, and long waiting times. The importance of multidisciplinary approaches and the need for systemic reforms to enhance service delivery were highlighted.

**Conclusion:**

Findings highlight a need for training programs for healthcare professionals to foster the values inherent to delivery of person-centered care.

**Supplementary Information:**

The online version contains supplementary material available at 10.1186/s12939-025-02408-3.

## Background

The escalating prevalence of dementia presents a substantial challenge, particularly in low- and middle-income countries (LMICs) such as India where 8.8 million people aged *≥* 60 years are estimated to be living with dementia [1]. As India’s population continues to age, this figure is expected to rise significantly in the coming decades [1–3]. Consequently, the need for efficacious dementia care is assuming greater importance. Unlike some developed nations with specialized dementia care services and comprehensive aged care systems, there are limited specialized services, diagnostic and treatment facilities, respite and long-term care options, and community-based programs in India, especially in rural areas and smaller cities and towns [4, 5]. Individuals diagnosed with dementia and their carers often rely heavily on the expertise and guidance provided by those specialist healthcare professionals they interact with, while seeking healthcare for dementia within hospitals, usually including neurologists, geriatricians, psychiatrists, neuropsychologists, general practitioners, nurse practitioners, physical/occupational therapists, nutritionists, and social workers [6–9]. In India, these diverse roles tend to be collectively referred to as ‘doctors’ by patients and families.

The quality of care these healthcare professionals provide is influenced by their interactions between individuals with dementia, family carers, and broader health and aged care systems [10, 11]. In the absence of specific dementia care services, these healthcare professionals provide not only medical care but also psychosocial guidance and support for individuals diagnosed with dementia and their carers [12]. Thus, the doctor-patient/family carer relationship becomes a critical factor influencing care quality [13]. Understanding these collaborative dynamics is crucial for enhancing the overall quality of care and ensuring a patient-centric approach [11, 12]. In this article, we describe the perspectives of carers and healthcare professionals regarding the attributes of a ‘good doctor’ in the context of accessing care for individuals with dementia in India in one large public hospital in Bengaluru, India. The term ‘good doctor’ is used to encompass not just medical practitioners, but the entire multidisciplinary team.

## Methods

This is a secondary analysis of data from the Moving Pictures India project, which used a mixed methods approach to develop and assess films and digital media aimed at enhancing dementia care in India [53]. The larger study consists of video interviews with family carers and healthcare professionals, stakeholder consultations, community surveys, a quasi-experimental trial, and evaluation of data analytics. In this article, we focus on the interview data with 19 family carers and 25 healthcare professionals. Data was collected in Bengaluru, India via semi-structured, face-to-face interviews conducted between March to July 2022.

### Participants and sampling

To be included, family carer interviewees were (a) actively involved in the care of a family member with dementia for a minimum of 6 months or had recent caring experience for deceased family member (< 2 years) with dementia, and (b) were proficient in English, Hindi, or Kannada. Healthcare professionals eligible for inclusion were those engaged in delivering health and community services to individuals living with dementia. Purposive sampling was applied to recruit carers from various genders, socioeconomic strata, and linguistic backgrounds. Sampling procedures in this study were purposive and iterative. The sample was determined by saturation– i.e., when no new information was emerging from interviews. The final sample is commensurate with similar studies on dementia care in India [14–17].

### Data collection

Patients and families at the outpatient department of a public hospital were invited to participate, along with healthcare professionals involved in dementia care in Bengaluru, India. Professionals were also asked to identify clients who might be interested in the video interviews. Potential participants were invited in-person or via email or telephone contact. Those who were interested then received the participant information and consent form, and following written informed consent, video interviews were scheduled at a time and place convenient for the participant. Interviews, conducted by RR, were video recorded with the help of a professional videographer.

Family carer interviews, in English, Hindi, or Kannada, focussed on dementia symptoms, caregiving, service collaboration, and care pathways. Healthcare professional interviews, conducted in English, centered on patient and family collaboration, care aspects, symptom management, advanced stages, and palliative care. The scope of the interviews for carers was to gather information about pathways to care, from diagnosis to meeting the increasing needs of individuals with dementia as the condition progressed. A specific section of the interviews explored carers’ experiences in providing care, the therapeutic relationships between carers and healthcare providers and attributes of a ‘good doctor’. Questions included:

- “Describe your experience interacting with healthcare professionals?” with prompts such as “Has your experience been positive or negative? How did it impact you? Do you have any suggestions for improvement?“.
- “How is your experience seeking hospital care? Can you comment on traveling, quality of assistance, cost, frequency of visits, and any challenges or unmet needs?“.
- “How did you choose your doctor?” with follow-up prompts like “Tell me if any healthcare professional stands out in your mind as exceptional and why? Describe the qualities of that provider. What made them stand out? What qualities do you consider important in your relationship with healthcare providers (e.g., nurse, GP, specialist, care attendant)?”

Broader insights, such as those related to healthcare policies, emerged naturally during discussions about systemic shortcomings and participants’ suggestions for improvement. The full interview guide has been included as Supplementary Material 1.

### Data analysis

Interview recordings were translated and transcribed into English and cross validated against the original audio recording for fidelity. Transcripts were de-identified, pseudonyms assigned to family carers, and then in NVivo version 12, a thematic data analysis was undertaken [18]. Independent coding by RR, JA and UB was completed, with a coding framework devised through multiple iterations and refinement of the codes. Senior investigators (BB, SL, CC, MV) scrutinized the entire analytical process to ensure methodological rigor, culminating in consensus-driven finalization of themes and sub-themes. The final schema was applied to the entire interview dataset.

### Ethical considerations

The study was approved by the NIMHANS and Curtin University Human Research Ethics Committees, the NARI Research Governance Office and the Health Ministry’s Screening Committee, India. The procedures outlined in this study are in accordance with the Declaration of Helsinki.

## Results

Participants characteristics are outlined in Table 1. There were 25 health professionals and 19 family carers; the latter were predominantly university educated, lived in an urban area, and had access to healthcare insurance. Four major themes were identified, each comprising several sub-themes that shed light on the participants’ experiences, perspectives, and recommendations regarding accessing dementia care and highlighting the qualities of a ‘good doctor’ (Table 2).

**Table 1:** Participant characteristics

Category	Family Carers (*n* = 19)	Healthcare Professionals (*n* = 25)
**Age (Mean ± SD)**	50.3 ± 15.2	39.86 ± 8.8
**Gender**	Women: 47.4% (*n* = 9)	Women: 76.0% (*n* = 19)
	Men: 52.6% (*n* = 10)	Men: 24.0% (*n* = 6)
**Relationship to person with dementia**	Adult Children: 57.9% (*n* = 11)	—
	Spouses: 31.6% (*n* = 6)	
	Others: 10.5% (*n* = 2)	
**Duration of Caregiving**	1–5 years: 47.4% (*n* = 9)	—
	> 5 years: 52.6% (*n* = 10)	
**Marital Status**	Married: 89.5% (*n* = 17)	—
	Single/Widowed: 10.5% (*n* = 2)	
**Religion**	Hindu: 73.7% (*n* = 14)	Hindu: 52.0% (*n* = 13)
	Muslim: 15.8% (*n* = 3)	Christian: 28.0% (*n* = 7)
	Others: 10.5% (*n* = 2)	Not disclosed: 20.0% (*n* = 5)
**Education Level**	High School: 10.5% (*n* = 2)	Bachelors/Diploma: 12.0% (*n* = 3)
	University: 89.5% (*n* = 17)	Masters/MPhil/PhD: 56.0% (*n* = 14)
		Medical Degrees: 32.0% (*n* = 8)
**Employment Status**	Full-time: 47.4% (*n* = 9)	—
	Part-time/Casual: 10.6% (*n* = 2)	—
	Homemaker: 15.8% (*n* = 3)	—
	Retired: 26.3% (*n* = 5)	—
**Residence**	Urban: 94.7% (*n* = 18)	—
	Rural: 5.3% (*n* = 1)	
**Access to Healthcare Insurance**	Government: 10.5% (*n* = 2)	—
	Private: 57.9% (*n* = 11)	
	None: 31.6% (*n* = 6)	
**Professional Roles**	—	Allied Professionals: 48.0% (*n* = 12)
		Specialists: 28.0% (*n* = 7)
		Others: 24.0% (*n* = 6)
**Tenure in Role (Mean ± SD)**	—	9.86 ± 7.4

**Table 2:** **Summarizing the themes**,** sub-themes**,** and codes from the thematic analysis**

Themes	Sub-themes	Codes
**Accessibility and Availability of the Healthcare Professional**	Barriers to Hospital Access	Physical accessibility challenges, long travel times, high costs of transport, long waiting times, difficulty navigating hospital environments, frequent rotation of doctors, high patient load, need for specialized treatment, limited support services.
	Facilitating Factors	Availability of facilities like canteens, open campus space, specialized clinics for older adults, prioritization in queues, staff efforts to reduce waiting times.
	Healthcare Professionals Adapting to Care Environment	Providing comprehensive evaluations, detailed consultations, sharing contact details, being available for consultations and follow-ups, remote consultations via online platforms, empathy in handling patient and carer needs.
**Empathetic Engagement and Effective Communication**	Comprehensive Evaluations and Detailed Consultations	Spending ample time with patients, understanding patient history, effective communication, and clear information sharing, providing regular feedback, attentive listening, being compassionate, fostering trust.
	Listening Skills	Patience, attentive listening, addressing concerns, explaining conditions in simple language, allocating time to carers and family members.
	Person-Centered Care	Treating patients with dignity and respect, recognizing emotional and psychological needs, encouraging autonomy, providing holistic and empathetic care.
**Knowledge and Competency**	Bridging Knowledge, Empathy, and Family Preparedness	Clear communication about dementia progression, setting realistic expectations, preparing families for future care needs, tailoring advice to individual circumstances.
	Adverse Clinical Experiences	Misdiagnosis, overmedication, costly and non-targeted interventions, prioritizing research over patient care, lack of support for carers, poor communication, and inadequate attention to patient needs.
	Positive Clinical Experiences	Competent multidisciplinary teams, comprehensive support, holistic approach, good facilities, empathetic healthcare professionals, staff collaboration, continuity of care.
**Systemic Reforms and Culturally Competent Multilevel Support**	Community-Based Initiatives and Policy Changes	Establishing more dementia care facilities, developing community care models, evidence-based interventions, barriers in help-seeking, culturally adapted assessments, training healthcare professionals, raising awareness, creating a National Dementia Action Plan, policy reforms.
	Recommendations for Healthcare Professionals	Honesty in communication, supporting carers, providing realistic prognosis, using clear language, fostering collaborative care, reducing barriers to accessing services.
	Recommendations for Healthcare System	Developing infrastructure, expanding service delivery beyond major cities, adopting a multidisciplinary approach, implementing early detection strategies, improving follow-up care, and increasing training and sensitization programs.

### Accessibility and availability of the healthcare professional

This theme explores the challenges and facilitators to accessing dementia care in India, specifically the difficulties faced by carers and persons with dementia in reaching and navigating busy hospital environments. Family carers talked about difficulties associated with accessing dementia-related healthcare as journeys to hospital could be lengthy due to distance and traffic.

*It is really a big challenge when transporting him [the person with dementia] to a hospital because… traffic*,* it might take one-two hours journey. So*,* managing two hours on road it is really*,* really hard. (Anand*,* man*,* 35*,* caring for his father)*

*It is expensive for the people who come from faraway places as it is difficult to bring dementia person in bus*,* and we had to depend on car or auto or cab to travel. And in the campus also*,* one has to walk long distance inside the campus from the gate to OPD [outpatient department] and many other departments. My mother would find it very difficult. (Sonia*,* woman*,* 35*,* caring for her mother)*

Prolonged waiting times within the hospital exacerbated distress and confusion experienced by people living with dementia. The substantial patient load encountered in tertiary healthcare facilities, frequent rotation of doctors and other healthcare professionals, coupled with the imperative to seek specialized treatment, were also identified as challenges.

*When we take the patients [to the hospital] there will be waiting period. Some time it would take two hours*,* three or four hours to meet the doctor. Till that [time] my mother would not sit patiently and handling her was a big challenge for us. (Shiva*,* man*,* 47*,* caring for his mother)*

Lengthy wait times were partially ameliorated by access to facilities such as canteens, green walking spaces within the hospital campus, and dedicated services for older adults.

*This [a public tertiary care hospital] is a big hospital*,* with so many patients*,* and thus it takes time. The facilities are good here. The canteen quality is good. Generally*,* we used to have lunch here*,* didn’t use to bring food from home. Doctors are good*,* the campus is spacious. We can take a walk. (Kunal*,* man*,* 34*,* caring for his mother)*

*In our clinic [geriatric clinic at a public tertiary hospital] we have separate OPD [outpatient] facility to diagnose the older people. They will be given priority in the queue. We try to attend them soon and provide a solution to their problem. Almost every hospital has this facility. (Psychiatrist*,* man*,* 14 years of experience)*

Within these challenging environments, healthcare professionals endeavoured to be accessible and available. Those who invested time in addressing the carer’s and family’s queries, and remained accessible for continued care and follow-up were valued.

*We used to call [the psychiatrist] on the phone and he’d always give me the time to talk to him to say what to do*,* and half an hour consultation. And he said*,* “Whenever you want you [can call]*,*” so that was great. He was a great support. (Nancy*,* woman*,* 65*,* caring for her husband)*

The emergence of online platforms post-COVID, enabling remote consultations was also recognized for enhancing accessibility to healthcare professionals, and so were the professionals who were willing to do remote consultations.

*Now [post-COVID] at least we can we have a lot of apps that we can depend on them*,* we can speak to the doctor*,* we can speak to them and get the medication based on a symptomatic treatment. (Anand*,* man*,* 35*,* caring for his father)*

### Empathetic engagement and effective communication

Active listening, clear and compassionate communication, and a tailored approach fostered support and satisfaction among carers regarding their doctor-patient relationship. Detailed assessments and comprehensive consultations were seen by carers as vital for understanding the patient’s history and current condition, which in turn facilitated personalized and effective care.

*The best thing here [geriatric clinic in a tertiary care hospital] I felt here that other hospitals may not have*,* is that from the first day*,* for every patient*,* they invest a minimum of 2–3 hours or more; so that they can understand a complete history of the patient and the current situation is assessed in detail.” (Kunal*,* man*,* 34*,* caring for his mother)*.

Effective communication, particularly attentive listening, was frequently highlighted as a critical attribute. Carers valued healthcare professionals who listened patiently, validated their concerns, provided information in simple language, and allocated sufficient time for carers to comprehend the information imparted, which helped in building trust and satisfaction. Lakshmi, a 71-year-old woman caring for her husband, said the following about the consulting psychiatrist, “*He [psychiatrist] talks with much concern (for patients and family). He listens to us carefully and consoles us. There we get some satisfaction. Doctor has to be like that only*”.

Participants also noted the importance of treating people with dementia with dignity and respect. Engaging with people with dementia and their families in a person-centered manner that acknowledged their emotional and psychological needs was seen as crucial.

*As a doctor you should impress upon them that you still have life. You still have the vitality in you to live*,* to experience the viability of life and the depth of life and depth of relationship. So that’s a huge challenge in India and many times people come with a lot of prejudice. So*,* one is how you deal with the person*,* how you deal with the people around that person is so very important and I tell them*,* “Make sure that he’s made to feel valued and valuable and restore the dignity that they have”. (Geriatrician*,* man*,* 20 years of experience)*

*Just because a person is not able to communicate*,* just because we are not able to reach the person*,* doesn’t mean that they don’t understand*,* they don’t feel. It is important that dignity of the person is maintained. (Psychiatric Social Worker*,* woman*,* 8 years of experience)*

### Knowledge and competency

Carers’ past experiences with misdiagnosis, inappropriate treatment, and unclear communication about the progression of their relative’s dementia, influenced their perceptions of their existing healthcare professionals’ knowledge and competence. Participants valued clear guidance and realistic expectations, provided sensitively, although preferences for direct communication varied.

*When you make a diagnosis if you don’t present it in a very holistic*,* optimistic*,* truthful way they [person with dementia] start assuming things and they would have seen somebody in advanced dementia where they lose navigation to go even go to the toilet*,* they can’t self-care. They immediately think*,* “I’ll become like that”. They may not reach to that but that’s what you harp into. So*,* I think it’s so very important that you take a patient step by step and you reveal the diagnosis much more gently than abruptly*,* take the family into confidence. (Geriatrician*,* man*,* 20 years of experience)*

Previous negative experiences such as doctors recommending expensive futile assessments, providing non-targeted interventions, prescribing strong medications, and prioritizing research over patient care, particularly in private hospitals, were perceived by carers as professional incompetence and eroded trust in the healthcare system.

*They are all doing only research with her. Not doing any medicine. ‘Done’ I said*,* ‘You didn’t give any medicine. Nothing is seen. Only you did your research work. And I am bringing her from a long distance*,* taking pains. (Pritam*,* man*,* 79*,* cared for his wife)*

*In one hospital*,* they gave high dosage medicine*,* when it became heavy of dosage*,* she (mother) forgot the remaining things also. Starting from there*,* that memory power did not come back at all. (Sudha*,* woman*,*39*,* caring for her mother)*

The holistic approach of multidisciplinary teams (MDTs) was repeatedly mentioned by participants, as an effective way to share care responsibilities and ensure that the person with dementia received comprehensive support that addressed their physical and emotional needs.

*When she was in the hospital*,* at that time*,* some of the nurses were excellent. They would come and interact with her and chat with her and try to make her cheerful. Then the physiotherapists were very good… I remember when for quite a few days she was not speaking at all and then when physiotherapy started*,* she suddenly started speaking. That and we’ve always had very good doctors. I’m extremely grateful for all that. (Kashish*,* woman*,* 63*,* cared for her mother)*

Carers recounted positive experiences with healthcare professionals, emphasizing instances where competent and supportive MDTs played a crucial role.

*They [the multidisciplinary treating team] conducted counselling for me and my mother when we came here. Because we did not know the full picture*,* sometimes we used to lose our temper as to how many times to repeat same thing. Our approach changed a bit after that [counseling]. Even now we may say some things*,* but my mother is fully prepared and answers him calmly*,* how much ever time he asks*,* because she was counseled in detail. Or else it would be difficult. (Sonia*,* woman*,* 35*,* caring for her father)*

Carers also appreciated the administrative staff for easing the challenges of navigating healthcare facilities.

*So*,* all of those things [challenges due to medical institutions not designed to permit easy access and navigation] were small*,* but they were very dysfunctional for me*,* because I had to take my husband along to make sure he’s not lost. We met one person behind the [reception/billing] counter who was like an angel godsend. He was really good. He explained what I had to do. (Nancy*,* woman*,* 65*,* caring for her husband)*

### Systemic reforms and culturally competent multilevel support

To enable healthcare professionals to provide better care, participants recommended systemic improvements such as the need for community-based supports beyond established tertiary specialist institutions, the establishment of more dementia care facilities, and the implementation of policies that support dementia care at a national level. The creation of a National Dementia Action Plan was seen as essential for improving care across the country.

*WHO has said that every country needs a National Dementia Action plan. We will need to move towards a dementia action plan… in India… where the populations are so large; I think policies will make a very big difference. (Neurologist*,* woman*,* 20 years of experience).*

Barriers to help-seeking, including fear of community judgment, cultural scepticism about biomedicine, reliance on alternative practices, and uncertainty about where to seek help, further highlight the need for healthcare professionals to build trust through culturally appropriate care.

*Many people say many things. Many have said to me also*,* that “Doctors are lying about dementia to make money. They [doctors] say dementia is a serious disease which needs to be treated lifelong*,* and they make money. Your mother is fine; we have seen her from childhood. Take her to that baba [holy man/traditional healer] than this doctor. Then*,* you have decided whether it is helpful or not.” Will taking to baba help or doctor? If you feel visiting baba will help*,* you can go*,* no wrong in that. We also visited a doctor for three years*,* but no improvement was seen. So*,* we changed the doctor. You also*,* if one baba is not helpful*,* can go to another. As the time passes*,* it [function] will not come back. I would like to say that. It is better if you understand early and make others also understand and start the work. (Shiva*,* man*,* 47*,* caring for his mother)*

Participants also advocated for comprehensive, culturally adapted, community-based initiatives such as the establishment of more specialized dementia care facilities across the country, along with evidence-based models of care.

*Yes*,* if they have something in communities which have activities and at that place*,* we have access to doctors or people who can address this situation then it may become easier. (Kashish*,* woman*,* 63*,* cared for her mother)*

*Also in community level*,* there is lack of culturally acceptable questionnaires*,* the existing instruments doesn’t pick up much of executive dysfunction and subtle problems which we usually seen in patients with dementia. So*,* we’ve to develop culturally sensitive and socially palatable kind of questionnaires (for screening and clinical examinations) which are kind of age and culture appropriate. (Psychiatrist*,* man*,* 10 years of experience)*

*We have to develop models of care*,* evidence based validated models of care that we can then replicate in every place. (Neurologist*,* woman*,* 20 years of experience)*

Healthcare professionals desired to do more for their patients by advocating for better infrastructure, including resources for training, day care centers, and other support services for individuals with dementia and their carers. Their comments reflect the interconnectedness of individual competencies and systemic supports in enhancing the overall quality of dementia care.

*In many cases I think to myself*,* patients need day care - where to send them? Apart from big cities like Bangalore*,* there are no easy arrangements for this. Some need to be hospitalized for respite*,* then there may be shortage of beds in big hospitals or lack of alternative arrangements. Thus*,* I feel that there is a need for improvement in several areas. (Psychiatrist*,* man*,* 14 years of experience)*

Sensitizing hospitals to the unique needs of dementia patients and their families, improving service delivery to reduce wait times, enhancing follow-up procedures, adopting a multidisciplinary approach to care, and increasing awareness and training on dementia for healthcare professionals and the community were other significant recommendations.

*Definitely I feel that every department in medical has to be trained on these areas*,* because nowadays cases are increasing. So*,* if a general medicine doctor doesn’t know how to deal with the dementia*,* so then it will be really challenging. So*,* it is good to have trained and at least basic knowledge*,* how to manage*,* what are the challenges*,* so that will really help them to do the things better. (Anand*,* man*,* 35*,* caring for his father)*

## Discussion

This study investigated carers’ and healthcare professionals’ perspectives on the attributes of a ‘good doctor’ in the context of dementia care in India. Central to our findings are the qualities both groups prioritize in defining a competent healthcare professional, particularly in accessing dementia care. Key attributes include accessibility to patients and families, empathy, effective communication, and demonstrable medical competence. These findings align with previous research that identified attentive listening, patient involvement in decision-making, and medical expertise as critical characteristics of a ‘good doctor’ [19–24].

Sharing personal contact details and willingness to spend time with the person with dementia and their family were considered hallmarks of a ‘good doctor’. Participants appreciated doctors who were accessible beyond regular working hours, a behavior described as ‘going out of the way.’ This finding resonates with a study among Norwegian hospital doctors, where being a ‘good doctor’ is linked to professional dedication, leading to increased efforts in workplace attendance and patient care effectiveness, regardless of circumstances [25]. However, it is noteworthy that while such gestures were voluntarily extended by healthcare professionals in the context of this study, they raise concerns regarding boundary challenges faced by healthcare professionals, including stress, burnout, and the potential for future boundary violations [26]. Given the high patient load and the shortage of professionals trained in dementia care in India, time is a luxury that may not always be readily available to doctors and other healthcare professionals [9]. It is worth considering whether the doctors’ inclination to take such actions reflects a deficiency in the community care safety net, possibly due to limited alternative options available to them.

Effective communication was also highlighted as important because understanding dementia progression and treatment options are crucial for empowering carers to make informed decisions. Poor communication and a lack of transparency can lead to feelings of disempowerment, exacerbated by medical paternalism and hierarchical structures within healthcare, which are pronounced in settings like India [27]. A ‘good doctor’ actively breaks down these barriers by ensuring clear, continuous information flow, empowering carers, and promoting collaborative care [11, 28, 29]. Communication training within medical education is increasingly recognized as fundamental to building trust and improving doctor-patient relationships [24].

In line with global trends toward patient-centered care, there is growing recognition of the importance of holistic, person-centered models [22, 30–32]. This shift mirrors changing perceptions of what constitutes ‘good’ care among Indian families. Earlier studies on dementia care in India indicated that families often equated care with the pursuit of a cure, investing significant time in seeking treatments and medications [16, 33]. However, the current study highlights a growing emphasis on spending time with the doctor and receiving counselling and information, suggesting a shift in families’ priorities. Professionals also demonstrate a more holistic understanding of the support needed. Despite these evolving perspectives, practical challenges remain, such as the chaotic nature of hospitals, long wait times, and limited accessibility, as noted by carers and healthcare professionals, all of which undermine the quality of care [34–36]. Addressing these issues requires improving service delivery, patient-friendly facility design, and reducing wait times, particularly for older people [28, 37, 38]. Remote consultations, which became more prevalent during the COVID-19 pandemic, were identified as a potential solution to some of these challenges [39].

Families valued a multidisciplinary, team-based care approach, which could facilitate the expansion of person-centered care in community settings. Initiatives like the *Moving Pictures India* project, for which these interviews were developed, aims to develop digital media resources to improve dementia care in India, so that good quality information and signposting can be widely available [53]. Goal-focused interventions, delivered by non-clinical support workers with training and supervision can help achieve personal goals for clients with dementia [40]. Expanding community care would optimize resource allocation by reserving specialized interventions for complex cases, while reducing the burden of travel and access barriers for those unable to reach clinics for logistical or financial reasons. This is particularly crucial in India, where community care remains the preferred option for supporting individuals, even with advanced dementia [41].

Ethical considerations also emerged as crucial to participants, especially regarding honesty in disclosing diagnoses and respecting cultural contexts. Negative experiences, such as prioritizing research over patient care or recommending expensive, unnecessary tests, highlighted the importance of upholding ethical standards in maintaining doctor-patient confidentiality and trust and ensuring patient autonomy [24, 42]. These ethical principles are central to improving the overall quality of care, particularly in resource-limited settings like India, where financial constraints often exacerbate feelings of helplessness.

Cultural nuances played a role in shaping dementia care and the perception of a “good doctor.” Cultural norms of family-oriented care placed the responsibility of caregiving primarily on family members, who may lack knowledge about the disease and access to formal care pathways [43, 44]. These dynamics influence the expectations from healthcare professionals, with carers valuing culturally sensitive support, clear communication, and empathy. Barriers to help-seeking, such as fear of community judgment, scepticism toward biomedicine, reliance on alternative practices, and uncertainty about accessing care, underscore the importance of healthcare professionals building trust through culturally sensitive approaches. A “good doctor” is expected to build trust by providing clear, culturally sensitive explanations of dementia, addressing misconceptions, and demonstrating transparency in treatment to counteract stigma and medical mistrust. Addressing these socio-cultural challenges is essential for designing effective dementia care policies and interventions, ensuring that carers’ needs are met while improving the accessibility and quality of care for individuals with dementia [43–45].

Participants’ recommendations focus on enhancing dementia care in India through community-based initiatives, policy-level changes, and increased awareness and training for healthcare professionals [9, 36, 46–48]. The call for a National Dementia Action Plan underscores the urgency of prioritizing dementia care on the national agenda, advocating for systemic reforms to improve service delivery, enhance carer support, and promote person-centered approaches [5, 32, 34].

### Limitations

This study has several limitations that must be considered. First, the sample, while reasonably heterogenous, were recruited from one large public hospital, and does not fully represent the diversity of carers and healthcare professionals across India. The urban focus may also limit application of the findings to other regions of India, particularly rural and underserved areas. While the hospital provided affordable, relatively well-structured and specialized dementia care services, such facilities are not universally accessible across the country. Access to this type of care is often constrained by socio-economic factors, geographic location, and healthcare infrastructure, which we could not explore comprehensively due to the urban-centric nature of our sampling. Second, nearly 90% of the family carers had a university-level education and access to healthcare insurance, which is not representative of the general population in India [49–51]. The study’s strengths are its rigorous qualitative approach, which enabled an in-depth exploration of the attributes of a ‘good doctor’ and the barriers and facilitators to accessing dementia care in India.

### Recommendations

Building on the findings from this study and insights from relevant literature, we propose a comprehensive plan for dementia care in India that addresses the critical challenges faced by individuals with dementia and their carers while emphasizing actionable steps for systemic improvement. A comprehensive plan for dementia care in India should focus on developing community-based support centers, raising awareness through culturally sensitive campaigns, and creating educational resources for carers [46]. Training programs for healthcare professionals and carers, alongside integrating dementia care into medical curricula, are essential to building capacity [9, 40, 44, 45]. MDTs must be promoted to ensure holistic care, with improved coordination between hospitals and community services. Policy reforms should prioritize a National Dementia Action Plan, expanded insurance coverage, increased funding for care facilities, and standardized care protocols [5, 34]. Technology, such as telemedicine and digital tools can bridge care gaps in rural areas [14, 39]. Culturally adapted interventions, including localized screening tools and traditional practices, are vital for patient-centered care [52]. Stakeholder engagement, collaboration with NGOs, and advocacy for dementia-friendly communities will further enhance support systems [34]. Monitoring and evaluating implemented programs will ensure continuous improvement and scalability to address dementia challenges effectively.

## Conclusion

The study offers valuable insights into the attributes of a ‘good doctor’ from the perspectives of family carers and healthcare professionals in India and identifies actionable strategies to improve dementia care within a multidisciplinary and patient-centered framework and enhance opportunities for patients and carers to experience good quality care. Future research should focus on incorporating diverse settings, including rural areas, to gain a deeper understanding of the intersectional challenges and disparities in accessing dementia care across India. Additionally, expanding the scope to explore cultural dimensions— how socio-cultural nuances shape expectations from healthcare professionals, including trust-building, culturally sensitive communication, and the need to address systemic and accessibility barriers —will provide valuable insights into the broader systemic and contextual factors influencing dementia care.

## Electronic supplementary material

Below is the link to the electronic supplementary material.

Supplementary Material 1

## Data Availability

Raw footage of the video interviews cannot be entirely de-identified and will not be publicly available. Other data set generated during this study, such as de-identified transcripts, will be available from the corresponding author upon reasonable request.

## Abbreviations

LMICs: low-income and middle-income countries
MDTs: multidisciplinary teams
