# Supplementary material for: Unveiling the qualities of a ‘good doctor’: family carers’ and healthcare professionals’ perspective on dementia healthcare in India
Source: Int J Equity Health. 2025 Feb 17;24:46. doi: 10.1186/s12939-025-02408-3 (PMC11834680; doi:10.1186/s12939-025-02408-3)
Supplement: Supplementary file 1 — Supplementary Material 1 [file 12939_2025_2408_MOESM1_ESM.docx]

Demographic information and Interview schedule

Carers

31 March 2020

Version 1

Participant name:___________________________________

Participant address & contact details: ___________________

_________________________________________________

Location/Time: _____________________________________

Interviewer: _______________________________________

| Participant demographic information | |
| --- | --- |
| Date | ____/____/____ |
| Gender |  |
| Age |  |
| Marital status | 🞏Married 🞏Single 🞏Divorced/Separated 🞏Widowed |
| Work status | 🞏Full time 🞏Part time 🞏Casual/Daily wager 🞏Unemployed  🞏Full time carer 🞏Homemaker 🞏Retired 🞏Student |
| Job |  |
| Level of education | 🞏None 🞏Primary school 🞏High school 🞏University 🞏Vocational |
| Number of years/Months as a carer |  |
| Religion |  |
| Languages spoken at HOMe |  |
| **Person with Dementia** |  |
| NAME |  |
| Gender |  |
| Age |  |
| Languages spoken at HOME |  |
| Religion |  |
| Marital status | 🞏Married 🞏Single 🞏Divorced/Separated 🞏Widowed |
| Level of education | 🞏None 🞏Primary school 🞏High school 🞏University 🞏Vocational |
| Current living situation | 🞏Living only with spouse 🞏Living only with children 🞏Living in nuclear family 🞏Living in joint family 🞏Supported living 🞏aged care 🡪if aged care, duration of stay: _____Months |
| Family support for person with dementia |  |
| NUMBER OF PERSONS IN THE household |  |
| NUMBER OF INDEPENDENT MEMBERS (HEALTHY ADULTS) |  |
| Number of Dependent members (Older people, persons with high support needs, and number of children) |  |
| NUMBER OF CAREGIVERS |  |
| NUMBER OF EARNING MEMBERS |  |
| ACCESS TO HEALTH INSURANCE | 🞏Government 🞏Private 🞏No Insurance |

| Carer interview guide |
| --- |
| Dementia; signs, symptoms, cultural idioms |
| - What problems did you first notice with regard to your___(relation to carer; ex: mother, father)____? - What was the person like before the illness/problem? - How have they changed? Has the illness/problem progressed, what were the various symptoms you have noticed? - When did you start to notice these changes? - What made you think something was wrong? (prompt events, symptoms) - What did you think was happening? - Tell me about what you initially did? |
| Dementia diagnosis |
| - Where did you seek help to start with?   - Who are the various service providers and professionals you visited and how was your experience?   - Did you try other avenues as well (other avenues of health care/traditional healing/elsewhere) (note: try to elicit the order of events)   - What was your _________diagnosed with?   - Who gave the diagnosis? - What did the doctor tell you? - How did you feel about hearing this information? - How did your __________ react? Did they understand the diagnosis/do they know they have dementia? Please elaborate. - How did other people around you react?   (Probe): have people’s reactions changed over time?   - What did you think about these reactions at the time? How do you feel about them now? - What is your understanding of what caused the dementia/ problem? Has your understanding of dementia changed since the diagnosis? - What do you feel about the information given to you when you received the diagnosis? - Did the information help you gain access to services/supports? Please explain - What was provided/what was lacking/what would you have liked more of? What information is important to get help? - Could you describe a day in the life of your______?   (Probe: activity level, the role of various family members throughout the day, interaction with outsiders,)   - What has worked for you and your _________?   - What do you do with your________ for fun?   - What does your______ enjoy doing?   - How has knowing your______ history helped you care for them?   - What sort of things does your______ like having nearby? - Is dementia something people talk about in your community?   - What is the general understanding of dementia in the community? Causes, how to manage, what symptoms are part of dementia?   - How do people interact with <name>/persons with dementia? Are people comfortable around <<name>>? |
| Care |
| - What sources of help have you used? - Other than health professionals, have you received support from anyone else? - Describe formal sources/services you have used-(Probe: Agency services: support or educational groups for carers /day-care/respite/ hired caretaker: trained?) - What have you found to be the most useful? - Was it difficult to know where to get help? Did someone help you (formal/informal)? - Can you describe any other sources of support received outside of formal care? - Can you describe the family support your_______ has in India? Who all are in the family, where do they live and how do they support your_______? - Please describe how you would help a friend who found themselves in a similar situation   - Where should they go?   - Who should they talk to?   - What should they look out for? How should they prepare? |
| Management and care |
| - What sort of treatments (if any) has your________ received? - What medications, psychological interventions (e.g. cognitive training) have been prescribed?   - Has the doctor suggested anything other than medications?   - What are the various activities your_______has been advised to do?   (Probe: memory exercises)   - Have they been effective? Please describe. - Have you been provided with any information/education on how to manage symptoms at home? - Who has been most helpful in providing information/education? (could be other carers, social networks etc.) - Are you and <<name>> using any strategies to manage the symptoms? Please describe (e.g. to deal with challenging behaviours, confusion, and communication difficulties). - Have there been times when your______couldn’t tell you what they needed, and you had to try and respond to their needs? Can you describe what happened and what you did in this situation? Do you have any tips that you use to respond to unmet needs? - How do you manage the care needs and do you have any tips that work for you;   - Related to nutrition and eating   - Dental care   - Bathing   - Toileting   - Incontinence/constipation   - General hygiene   - Pain   (Do you have any concerns regarding these?)   - What have your health professionals informed you about future care needs of your________ and challenges? - Have you had to plan palliative care/end of life care? If so can you describe the process? Did you have any support? Formal services? Family and friends? - How has your experience of providing care to your________ been? - Are there any skills you have needed to learn to manage? - As a carer, have you sought support for yourself?   - Have you been encouraged to seek care for yourself by health professional/service provider? - Please describe if you have sought support and why you felt this was important (carers groups, education, emotional, physical or social support from formal/informal avenues, financial considerations, carers allowance?) |
| Therapeutic relationship |
| - With relation to dementia can you describe your experience interacting with health care professionals? - Has your experience of engaging with services been positive or negative? -how so? How did it impact you? Suggestions for improvement? - How is your experience seeking hospital care? Can you comment about traveling, quality of assistance, cost, frequency of visits, challenges/unmet needs if any? - How did you choose your doctor?   - Tell me if any one health professional stands out in your mind as exceptional   - Why? Describe the qualities of that provider? What made them stand out   - What are some of the qualities you consider important in your relationship with health care providers? (prompts: nurse, GP, specialist, care attendant, others?) - Have you had instances where you had difficulty following the doctor’s advice?   - Have you ever received conflicting advice from different people? What did you do? - Where the wishes & needs of <<name>> and you considered in the planning of dementia care?     Do you have any other comments? |
| Care amidst COVID |
| Did the Covid pandemic impact the care that your_____ received? How so? How did the past few years differ from pre-pandemic?  (Prompts; Formal services; access to services, help at home? Not being able to see providers etc.. Informal services; isolation, was it harder for the carer)  What helped you overcome these challenges?  (Prompts; any resources/services you used? Formal services, informal services, help form the community; use of technology)  Anything that you will keep doing now there are no restrictions in place? Please explain. |

Demographic information and Interview schedule

Health Professional

| PARTICIPANT DETAILS |  |
| --- | --- |
| Name & title |  |
| Age |  |
| Gender |  |
| Phone # |  |
| Email |  |
| Languages spoken |  |
| Cultural Background |  |
| Qualifications |  |
| Role |  |
| Number of years in role |  |
| Organization & address |  |
| Exact name, title and organizational name for film credits [*if possible obtain business card*] |  |
| Other details if needed |  |
| INTERVIEW DETAILS |  |
| Date and time |  |
| Location |  |
| Interviewer |  |
| Interview language |  |
| Reminder call date |  |

| Health Professionals interview schedule |
| --- |
| Service provider background   - Can you tell me a bit about your role? How long have you been working in this field? - How often do you see people with dementia? - For the sake of explaining to the audience, could you explain the common symptoms of Dementia? What is the difference between memory decline because of normal aging and memory loss due to dementia?   Causes and characteristics   - Can you tell me the process of diagnosis for your patients? How do families/people with dementia describe their symptoms? - At what stage of illness do people usually present to you? Delays? Can you think of reasons for delayed presentations/delays in help-seeking? - What symptoms of dementia do people overlook? Do they commonly see you with concerns of dementia, or do they come for other reasons (please explore)? - Apart from not recognising symptoms early enough, what are some of the barriers people face in receiving a timely diagnosis and accessing appropriate interventions? - What cultural beliefs do you think may contribute to symptoms being overlooked or ignored? - What are some of the misconceptions that family members may have about dementia when their loved one is first diagnosed? - (If medical professional who conveys diagnosis) What do you tell families when you convey the diagnosis (types of dementia, causes and brain changes, cure, management, suggestions for forward planning) - How do families and/or people with dementia respond to diagnosis, and the prognosis? What interventions or services are available (at your organisation) to help them during this initial process?   Risks and health promotion   - What are the benefits of early diagnosis? - (if health professional) Can you describe what some of the protective factors for dementia are? What are some of the risk factors?/ Do you share information about protective factors with the family, if yes, what? - How do health professionals promote the general health of persons with dementia? What do you particularly inform the family and the persons with dementia? (*environment care setting, activity level of individual, number of care providers,)*   Communication and behaviour   - How can families best engage with doctors and health staff? Are there some important points people should consider before their visits (prompts; prepare questions? Be prepared to ask advice from the doctors etc) - How do you best communicate with a person with dementia? Give examples if possible/ are there any guidelines do’s and don’ts (golden rules)?   - Other than verbal communication, are other aspects of communication important (e.g. touch, body language)? - Can persons with dementia feel and communicate pain? What are the causes for pain? How is pain managed for persons with dementia?   Care considerations and Pathways of care   - What sorts of medications/interventions/referrals do you usually suggest? Could you please explain why? - What is the role of medications? How effective are medications? - Apart from medications, what else can be done to manage symptoms of dementia? - Do you provide families/carers/persons with dementia information about the management of symptoms? If so, what information do you share/ what is your most common advice? - What are the key issues that families report to you? Then what do you do? What advice do you give them? - Do you know what other health practitioners, families with a person with dementia have seen prior to seeing you? Do you know what the care trajectory looks like? Does it differ across cultures? Why? - Do you think families and people with dementia seek alternative treatments? What sorts? What do you think about that? - What do people need to realise about caring for someone with dementia? - What are the present shortcomings in the health/care system for people with dementia and their families? What do you think needs to be done in your profession to improve things? - What sort of things need to be done more broadly to improve support to people with dementia and their families?   Prompts: Health literacy? Language? Structural barriers?   - How do you help your clients plan for the progression of the disease?   - What practical advice do you give about how to care at home? what do you feel people need to know about:     - Personal care such as hygiene: dental care, toileting, incontinence and other ‘bath and body’? (what do you feel carers need to know, how would you explain it)     - Managing difficult behaviours? –give examples (what do you feel carers need to know, how would you explain it)       - In your experience, what are some of the common changes in behaviour in a person with dementia?       - Are there any difficult behaviours that are commonly noted? Could you elaborate on few?       - What may underpin difficult or aggressive behaviours? (hint: Pain, unmet needs)       - Does the environment influence behaviours? (if so how)       - What do you feel carers need to know about managing difficult behaviours, how would you explain it?     - Eating and nutrition (what do you feel carers need to know, how would you explain it)     - Recognising and managing unmet needs in person with dementia? (give examples and explain how you might manage) (What are the various unmet needs of persons with dementia? How can one recognise and manage? How do you manage it? What do carers need to know?) - What are the needs of someone with dementia at different stages? - What is end of life/palliative care for persons with dementia? How do you navigate discussion about the same with the family? What advice do you give people about end of life care? Is this something that people consider? Why/why not - How to families and carers respond? What are their needs at this stage? - What do you feel carers need to know? (Prompt: Can you explain, if you do this in your work, what you recommend families and clients to plan ahead? (hint: Legal: power of attorney, advanced care directives, wills. Care: care planning, respite care, in-home care, residential care: CARER support?)   Stigma, communication and behaviour   - How do you think the public perceives dementia? What do they think of the family touched by dementia? - How does the public perception of dementia affect the families you see? (Give some examples) - What are some of the misconceptions you think people in the community may have about people with dementia? Are there differences across cultures and community groups about how to interact with someone with dementia?   Carers   - What are some of the things carers need to consider in their daily lives? What are the various unmet needs and challenges of the caregivers? Do you think it is important for carers have breaks from caring (why/why not?) - How is the quality of life of carers, you encounter in your practice? What are your recommendations to carers, to maintain a balance in their lives? How can carers better take care of themselves? (Prompt- self care, taking time out, sharing the care? ) - What do you recommend carers do to take care of themselves? What are some of the barriers to carer self care? (expectations, gender roles, cultural understandings of care. Practical issues such as money, access and or availability of services/ others in family? ) - What resources are available to carers? What sort of initiatives are currently available and what would you like to see more of? - How do you think community members/groups could help/ come together to support people with dementia and their carers/families?   General   - Any other comments you would like to make?   Thank you for your time |
